# Application of the UHPLC-DIA-HRMS Method for Determination of Cheese Peptides

**DOI:** 10.3390/foods9080979

**Published:** 2020-07-23

**Authors:** Georg Arju, Anastassia Taivosalo, Dmitri Pismennoi, Taivo Lints, Raivo Vilu, Zanda Daneberga, Svetlana Vorslova, Risto Renkonen, Sakari Joenvaara

**Affiliations:** 1 Department of Chemistry and Biotechnology, School of Science, Tallinn University of Technology, Ehitajate tee 5, 12616 Tallinn, Estonia; dmitri@tftak.eu (D.P.); taivo.lints@tftak.eu (T.L.); 2 Center of Food and Fermentation Technologies, Akadeemia tee 15A, 12618 Tallinn, Estonia; anastassia@tftak.eu (A.T.); raivo@tftak.eu (R.V.); 3 Institute of Oncology, Riga Stradins University, 13 Pilsonu Str., LV-1002 Riga, Latvia; zanda.daneberga@rsu.lv (Z.D.); svetlana.vorslova@rsu.lv (S.V.); 4 Transplantation Laboratory, Haartman Institute, University of Helsinki, FI-00014 Helsinki, Finland; risto.renkonen@helsinki.fi (R.R.); sakari.joenvaara@helsinki.fi (S.J.); 5 HUSLAB, Helsinki University Hospital, FI-00029 Helsinki, Finland

**Keywords:** dairy product analysis, cheese peptidomics, cheesemaking, data-independent acquisition

## Abstract

Until now, cheese peptidomics approaches have been criticised for their lower throughput. Namely, analytical gradients that are most commonly used for mass spectrometric detection are usually over 60 or even 120 min. We developed a cheese peptide mapping method using nano ultra-high-performance chromatography data-independent acquisition high-resolution mass spectrometry (nanoUHPLC-DIA-HRMS) with a chromatographic gradient of 40 min. The 40 min gradient did not show any sign of compromise in milk protein coverage compared to 60 and 120 min methods, providing the next step towards achieving higher-throughput analysis. Top 150 most abundant peptides passing selection criteria across all samples were cross-referenced with work from other publications and a good correlation between the results was found. To achieve even faster sample turnaround enhanced DIA methods should be considered for future peptidomics applications.

## 1. Introduction

During cheese ripening, caseins undergo a progressive breakdown by enzymatic action, releasing peptides and amino acids, which contributes to the development of cheese flavour and texture [1]. The term “peptidomics” has been extensively used in dairy science for comprehensive analysis of peptides released during proteolysis in different cheese varieties [2,3,4] as well as characterisation of bioactive peptides with potential nutritional and health-promoting effects [5,6]. Several researchers have been focusing on the identification of phosphorylated peptides [7] and the determination of specific bitter peptides and their contribution to cheese flavour [8,9]. Many studies have been carried out to evaluate the effect of different adjunct cultures on the formation of peptides in cheese and thus to adjust the taste and aroma characteristics of a final product [10,11].

The key analytical tool employed in cheese peptidome research (i.e., increasing the knowledge of proteolytic events occurring during ripening) as well as exploring the possibilities of controlling the cheese maturation process, is currently mass spectrometry (MS) coupled with liquid chromatography (LC) [12,13,14]. The most widely used hyphenation is nano ultra (high) performance liquid chromatography (nanoUHPLC) coupled with high-resolution mass spectrometry (HRMS) based on data-dependent acquisition (DDA) mode [15,16,17].

The samples complexity combined with the slow acquisition rate of DDA modes as well as the ever-growing demand for higher protein coverage typically results in analytical gradients that exceed 60 or even 120 min, making such methods less appealing for higher-throughput studies [4,14]. Data-independent acquisition (DIA) is an alternative acquisition mode to DDA. DIA, unlike DDA, does not rely on precursor isolation. DIA is based on a principle of a rapid alternation between low and high collision energies to acquire MS^1^ and MS^2^ spectra. DIA relies on a chromatographic alignment of MS^1^ and MS^2^ for fragment-precursor assignment. Operating at higher acquisition rates and being compatible with faster gradients, DIA has been employed in several food research applications such as food safety, authenticity testing and peptide profiling of various food matrices [18,19]. Using DIA it is possible to simultaneously acquire both qualitative and quantitative data.

The aim of this study was to develop an LC-DIA-MS-based methodology for cheese peptide profiling with a sub-60 min analytical gradient without compromises in chromatographic performance and protein coverage.

## 2. Materials and Methods

### 2.1. Materials

Hi3 EColi STD (p/n: 186006012) and [Glu1]-Fibrinopeptide (p/n: 700004729) were purchased from Waters Corporation (Milford, MA, USA). Peptide quantitation was performed using Pierce™ Quantitative Colorimetric Peptide Assay (C/N: 23275, Thermo Fisher Scientific, Waltham, MA, USA). Nanosep^®^ Centrifugal Devices with Omega^™^ membrane 3 K were obtained from Pall (p/n: OD003C34, Port Washington, NY USA). Ultrapure water (18.2 MΩ.cm) was prepared with MilliQ^®^ Direct-Q^®^ UV (Merck KGaA, Darmstadt, Germany). Acetonitrile (MeCN; LiChrosolv, hypergrade for LC-MS,) and formic acid (FA; LC-MS grade) were acquired from Sigma-Aldrich (Darmstadt, Germany).

### 2.2. Cheese Manufacture and Sampling

Three Gouda-type cheeses (Cheese 1, Cheese 2 and Cheese 3) were produced using three different DL-starter cultures (DL1 and DL2 by Chr. Hansen Ltd., Hørsholm, Denmark and DL3 by DuPont^™^ Danisco^®^, Copenhagen, Denmark) at a dairy plant from 600 L of pasteurised (at 74 °C for 15 s) milk. Animal rennet (25 mL 100 1/L; 230 IMCU 1/g; 20/80) of chymosin and bovine pepsin, (Chr. Hansen Ltd., Hørsholm, Denmark) was added to milk. After coagulation, the curd was cut, whey removed, and cheese grains stirred and heated at 32 °C for 30 min. Cheeses were prepressed under whey, moulded, pressed for 1.5 h, brine salted (pH 5.1) for 36 h, waxed and ripened at 10–15 °C for 90 days. Samples were taken from each cheese at 0 (after salting), 14, 30 and 90 days of ripening, grated and stored at −20 °C for further analysis.

### 2.3. Sample Preparation

To prepare water-soluble extracts (WSE) of cheeses, 2.5 g of grated cheese was homogenised in 22.5 mL of MilliQ^®^ water (12,500–13,000 rpm) using Polytron PT 2100 dispersing aggregate with a diameter of 20 mm (Kinematica AG, Switzerland). Samples were heated for 10 min at 75 °C and centrifuged for 20 min at 4 °C at 13,302 g. Supernatants were stored in 1.5 mL Eppendorf^®^ Protein LoBind microcentrifuge tubes (Eppendorf AG, Germany) at −20 °C until further purification. Thawed sample supernatants (250 µL) and MilliQ^®^ water (250 µL) were transferred into the Eppendorf^®^ tube and vortexed for 30 s. Obtained mixture (400 µL) was added to Nanosep^®^ with 3 K Omega^™^ spin filter. Samples were centrifuged at 11,200 g for 15 min. For peptide quantification 30 µL of filtrate was mixed with 970 µL of working reagent. After 30 min incubation at room temperature, absorbance was measured at 480 nm (Helios Gamma, Thermo Electron Corporation, Waltham, MA, USA) and concentrations calculated using a blank-adjusted calibration curve. For HRMS analysis, 30 µL of filtrate was mixed with 970 µL of MilliQ^®^ water (1% MeCN and 0.1% FA). Using results from the peptide concentration measurement samples were further diluted to result in 100 ng/µL equalising column load across all the samples. The sample (195 µL) was transferred into a vial and spiked with 5 µL of 1 pmol/µL of Hi3 EColi STD.

### 2.4. Liquid Chromatography Mass Spectrometry

Samples were analysed using Waters nanoAcquity UPLC^®^ system (Waters Corporation, Milford, MA, USA) coupled with a Waters MALDI SYNAPT G2-Si Mass Spectrometer equipped with NanoLockSpray Exact Mass Ionisation Source and controlled by Waters MassLynx 4.1 (V4.1 SCN916, Waters Corporation, Milford, MA). Mobile phases were as follows: (A) MilliQ^®^ + 0.1% formic acid and (B) MeCN + 0.1% formic acid. Injection volume was 2 µL. Samples were loaded onto Acquity UPLC^®^ Symmetry C18 Nanoacquity 10 k 2 g V/M Trap column (100A, 5 µm, 180 µm × 20 mm, Waters Corporation, Milford, MA, USA). Loading was carried out for 1 min at 5 µL/min using 1% B. Loaded sample was further analysed using Acquity UPLC^®^ M-Class HSS T3 Column (1.8 µm, 75 µm × 150 mm, Waters Corporation, Milford, MA, USA) kept at 40 °C. The gradient was as follows: 0–1 min hold at 1% B, 1–10 min linear gradient 1–15% B, 10–40 min linear gradient 15–35% B, 40–45 min linear gradient 35–95% B, 45–53 min hold at 95% B, 53–55 min linear gradient 95–1% B, 55–70 min hold at 1% B. Analytical flow rate was 0.3 µL/min.

The instrument was operated in positive polarity and resolution mode (35000 FWHM at 785.8426 *m/z*). Data were acquired in MS^E^ mode with a scan time of 0.5 s between 1 and 50 min. Recorded mass range was from 50 to 2000 *m/z* for both low and high energy spectra. The collision energy was ramped from 15 to 45 V in the trap cell of the instrument. Cone voltage was set to 40 V and capillary voltage was set to 2.4 kV. Source offset was 60, source temperature was 80 °C. Cone gas was 50 L/h, nano flow gas was 0.3 bar and purge gas was 100 L/h. [Glu1]-Fibrinopeptide was used as LockMass for mass axis correction and was acquired every 30 s.

### 2.5. Raw Data Processing

The raw data files were imported to the Progenesis QI for proteomics software (Nonlinear Dynamics, Newcastle, UK). During the import masses were lock mass corrected with 785.8426 *m/z*, corresponding to doubly charged [Glu1]-Fibrinopeptide B. Default parameters for peak picking and alignment algorithm were used.

The peptides were searched against beta-casein (β-CN; P02666), alpha _s1_-casein (α_s1_-CN; P02662) and alpha _s2_-casein (α_s2_-CN; P02663) sequences from bovine species obtained with the UniProt database [20].

The protein identifications were done against sequences added with a spike in Hi3 standard ClpB protein sequence, CLPB_ECOLI (P63285). Nonspecific cleavage was chosen and zero missed cleavages were allowed. Fragment and peptide error tolerances were set to auto and false discovery rate to <1%. One or more fragment ions per peptide were required for ion matching. The following variable post-translational modifications were used in the analysis: oxidation (M), acetyl-(protein N-terminal), deamidation (NQ) and phosphorylation (STY). The analysis of each post-translational modification was done separately, and the results were combined. Absolute mass error for a peptide was set to 5 ppm and we included peptides with one to three charges in the analysis. In the sample grouping, the within-subject design was used, fold changes and repeated measures of ANOVA were used for statistics. Filtered data were exported and then subjected to the normalisation of peptide abundances based on the coefficients of each sample dilution.

### 2.6. Data Analysis

An additional batch of samples was analysed using the methodology described by Taivosalo et al. [14] to highlight differences in results between two approaches. DDA experiment raw data files were imported to MaxQuant proteomics software (https://www.maxquant.org/) for data analysis as described in the publication and subsequently exported for intramethod correlation analysis. Filtered data were exported and then subjected to the normalisation of peptides abundances based on the coefficients of each sample dilution. Normalised abundances were used to construct a data matrix to identify differences between sample peptide compositions.

The comparison of the DIA and DDA methods was done with the help of in-house data analysis and visualisation scripts written in the Python^™^ programming language (Python Software Foundation). For both methods for each measured sample, the top 150 peptides with the highest intensities were found. The locations of those peptides were then found on the protein sequences the peptides originated from, and peptide coverage profiles were created for each casein in every sample for both methods, showing the peptides coloured by the intensity and laid out on their corresponding protein sequences.

## 3. Results

Overall, 558 peptides were identified among the analysed samples across 90 days of ripening using our method. The variation in peptide profiles and abundances was evaluated.

Ten per cent of samples (Day 0 of each cheese) were injected as triplicates. Relative standard deviation for all replicates equated to 10.88%.

**Figure 1:**
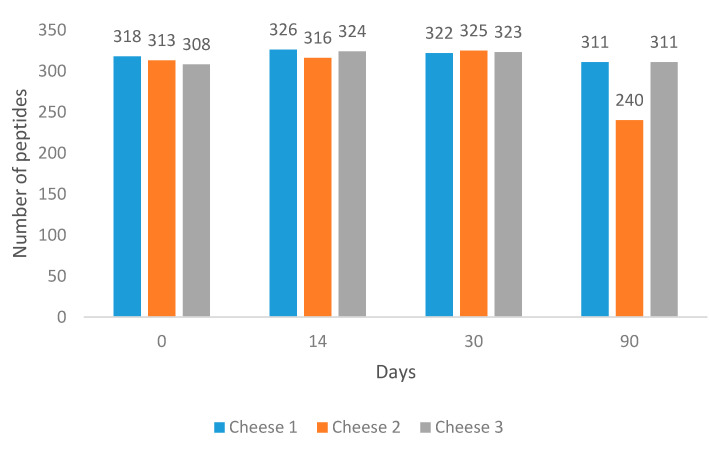
Number of identified peptides with unique amino acid sequences across 90 days of ripening.

It was found that at Day 90, Cheese 2 had the lowest number of peptides (Figure 1).

Cheese 1 and Cheese 3 had 6.7- and 6-times higher summed peptide intensities compared to Cheese 2 (Figure S1).

**Figure 2:**
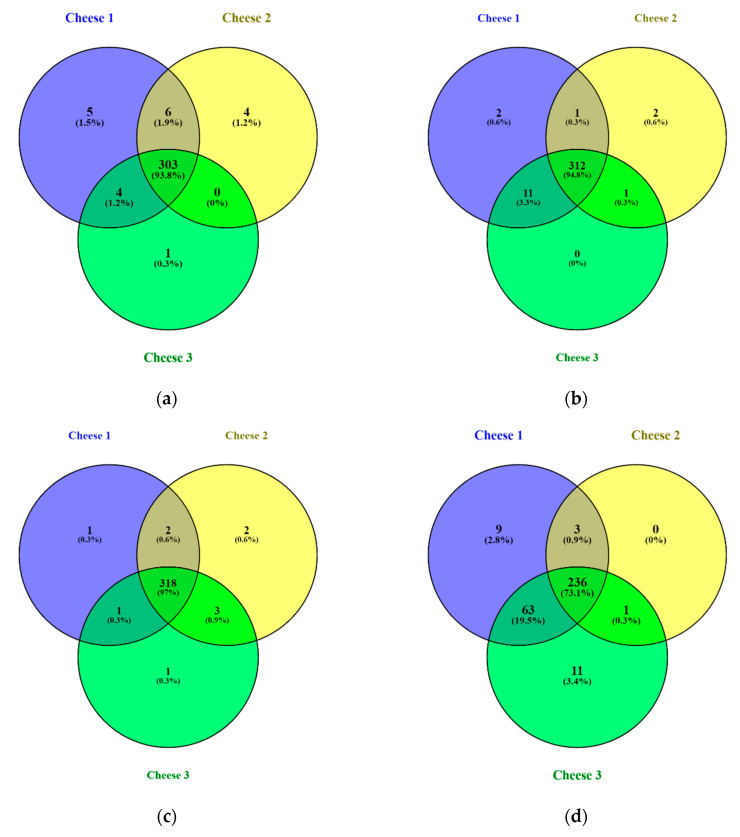
Venn diagrams of peptide distribution for Cheese 1, 2 and 3: (**a**) 0 days of ripening, (**b**) 14 days of ripening, (**c**) 30 days of ripening, (**d**) 90 days of ripening. Percentages in brackets denote proportion of all identified peptides across all cheeses.

At the same time, the average length of peptides in Cheese 2 was found to be longer than in other cheeses (Figure S2). All cheeses were subjected to comparative analysis to identify unique peptides at each measured point during cheese ripening. Results of the comparison are illustrated in Figure 2 that displays four Venn diagrams [21] for different days of ripening.

It was found that identified peptides during the first month of ripening were highly similar and accounted for approximately 93% identified peptides in all samples. During the ripening process, similarities in peptide composition between cheeses started to decrease. At the 90th day of ripening, Cheese 1 and Cheese 3 were more similar in peptide composition compared to Cheese 2, including over 60 identified peptides that were not present in Cheese 2.

**Figure 3:**
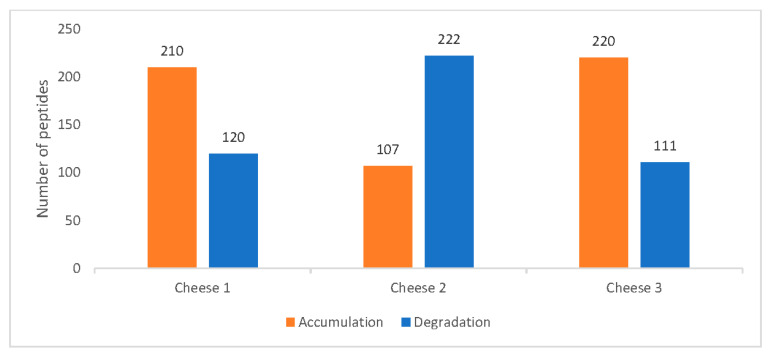
Cheese peptide profile trends between Days 0 and 90.

Figure 3 indicates the difference in peptide accumulation and degradation pattern between days 0 and 90 for all three cheeses. Cheese 2, unlike the other two, displays the prevalence of peptide degradation compared to accumulation. This pattern is also consistent with the summed peptide intensities of each sample.

**Figure 4:**
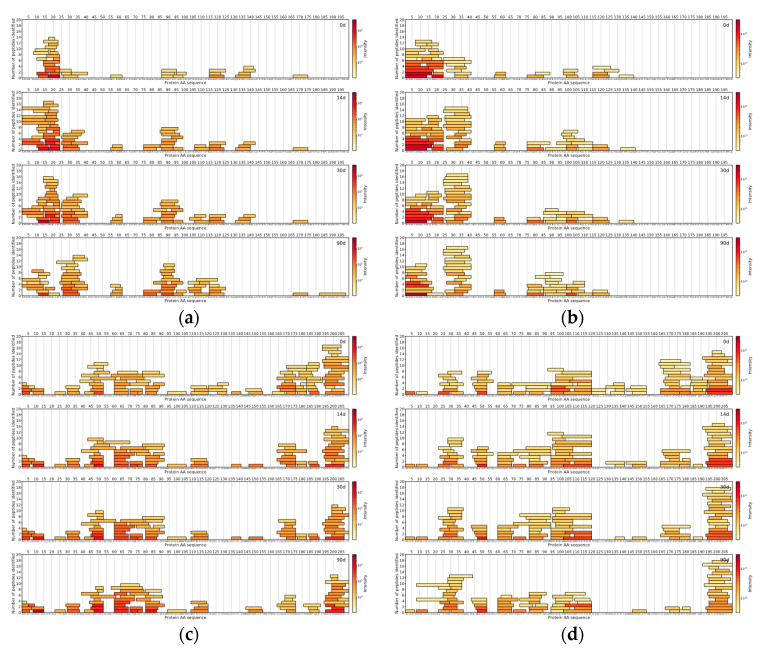
Cheese 1 α_s1_-CN: (**a**) data-independent acquisition (DIA)-MS and (**b**) data-dependent acquisition (DDA)-MS. Cheese 1 β-CN: (**c**) DIA-MS and (**d**) DDA-MS. The *X*-axis represents the casein amino acid sequence, the *Y*-axis represents a number of peptides and colour represents the logarithmically scaled intensity of peptides.

For comparison between DDA and DIA-based approaches, the top 150 most abundant peptides per method were selected by their normalised intensities at the 90th day of ripening. It was found that 70 unique peptide sequences with a median length of nine amino acids were similar between DIA and DDA approaches, based on the identified peptides from Cheese 1. DIA-based approach results showed 80 unique peptide sequences with a median length of a peptide of seven amino acids. On the other hand, the DDA-based approach results showed 80 unique peptide sequences with a median length of 10 amino acids (Figure 4).

**Figure 5:**
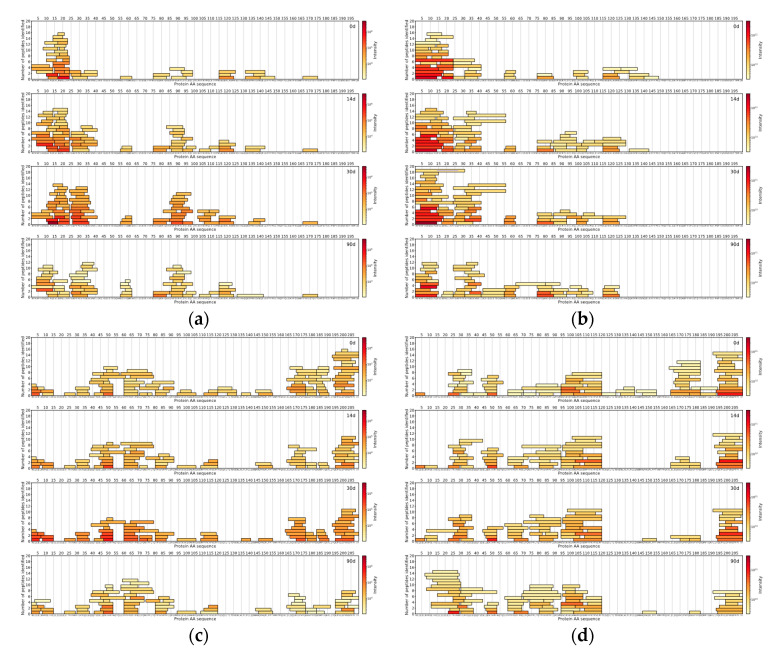
Cheese 2 α_s1_-CN: (**a**) DIA-MS and (**b**) DDA-MS. Cheese 2 β-CN: (**c**) DIA-MS and (**d**) DDA-MS. The *X*-axis represents the casein amino acid sequence, the *Y*-axis represents a number of peptides and colour represents the logarithmically scaled intensity of peptides.

As for peptides identified in Cheese 2 sample, 58 unique peptides sequences (median length: 9 AA) were common between two methodologies, 92 unique peptide sequences (median length: 7 AA) belonged to DIA-based approach results and 92 unique peptides sequences (median length: 10 AA) were found only in the DDA-based approach results (Figure 5).

**Figure 6:**
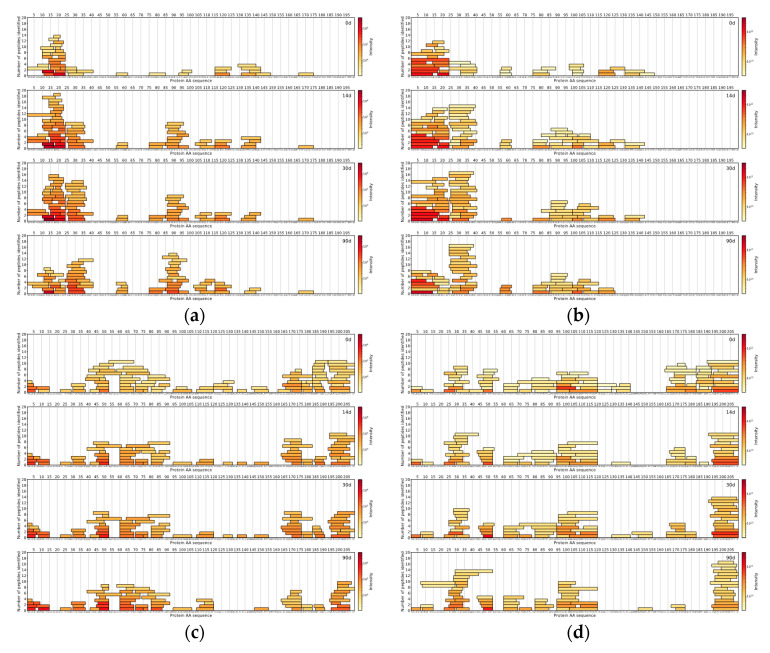
Cheese 3 α_s1_-CN: (**a**) DIA-MS and (**b**) DDA-MS. Cheese 3 β-CN: (**c**) DIA-MS and (**d**) DDA-MS. The *X*-axis represents the casein amino acid sequence, the *Y*-axis represents a number of peptides and colour represents the logarithmically scaled intensity of peptides.

Peptides identified in Cheese 3 showed a similar trend as 74 unique peptide sequences (median length: 8 AA) were in common between two methodologies, 76 unique peptide sequences (median length: 7 AA) were found in DIA-based approach results and 76 unique peptide sequences (median length: 11 AA) belonged to the DDA-based approach (Figure 6).

Across all samples analysed with either DIA- or DDA-based approaches, peptides from α_s1_-CN and β-CN comprised the majority of all detected peptides in the top 150 most abundant peptides.

In this study, we have also found several peptides, that have been previously reported to show bioactivity [6,22]: VPITPT (α_s2_-CN f117-122), MPFPKYPVEPF (β-CN f109-119), EPVLGPVRGPFP (β-CN f195-206), DKIHPF (β-CN f47-52), YPFPGPIPN (β-CN f60-68), TPVVVPPFLQPE (β-CN f80-91), VPGEIVE (β-CN f8-14), VPSERYL (α_s1_-CN f86-92), VLGPVRGPFP (β-CN f197-206) and YPFPGPI (β-CN f60-66).

## 4. Discussion

**Figure 7:**
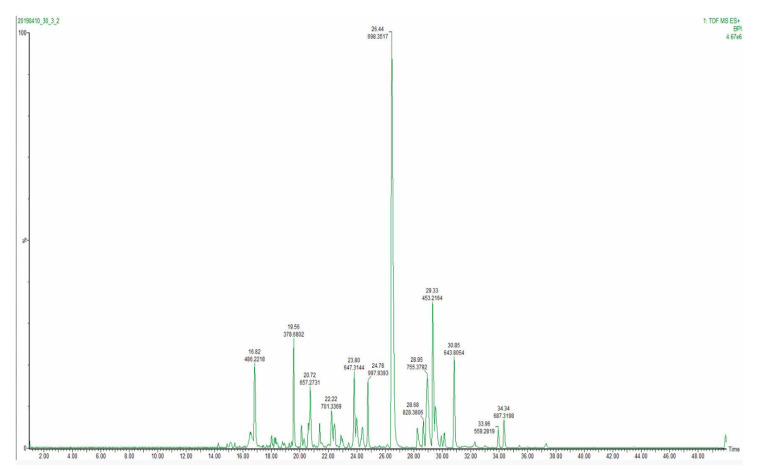
Base peak intensity (BPI) chromatogram of 40 min analytical gradient.

During chromatographic method development, three peptide elution profiles were evaluated. The separation was performed with 40, 60 and 120 min analytical gradients to compare chromatographic performances and MS method compatibility. Figure 7 displays a base peak intensity chromatogram for a 40 min analytical gradient method. The narrowest extracted peak chromatogram was at 10 s at the base of the peak, providing a sufficient number of data points per peak (Figure S3). Therefore, as 60 and 120 min methods did not result in a higher number of identified peptides, a 40 min analytical gradient was selected as the one facilitating the best throughput.

Although the conventional DDA approach provides cleaner MS^2^ spectra due to active isolation of the precursor, it suffers from a phenomenon known as data completeness problem [19]. While most abundant species get their corresponding MS^2^ spectra recorded, less abundant species can potentially be missed. Due to sample-to-sample variation in analyte concentration in combination with precursor selection criteria even a peptide eluting at the same time might be missed out.

DIA is not only not subjected to the aforementioned drawback of DDA, but it also operates at a significantly higher acquisition speed due to minimising the time between MS^1^ and MS^2^ scan acquisition. Therefore, faster acquisition rate does not only allow to record data qualitatively, but also quantitatively. However, DIA-based methods with a quadrupole set for static transition exceedingly rely on chromatographic separation to minimise peptide coelution and hence, acquire cleaner MS^2^ spectra.

Furthermore, during synthesis in the mammary gland, caseins undergo post-translational changes in their primary structure [23]. One of the most important post-translational modifications in caseins is phosphorylation (at Ser, Thr and Tyr residues) and thus, analysis of phosphorylated peptides requires additional enrichment and purification step to decrease ion ionisation competition between non- and phosphorylated peptides [24]. With the current method, it is not possible to robustly analyse the peptides with every possible modification.

In recent years methods of further enhancement of a conventional DIA approach are gaining significant popularity. Active scanning (SONAR^®^, Waters and Scanning SWATH^®^) or stepped (SWATH^®^, Sciex) quadrupole and ion mobility separation (HDMS^E®^, Waters and PASEF^®^, Bruker)-based DIA methods further expand the capabilities of DIA [25]. Active scanning or stepping quadrupole-based DIA methods significantly improve spectral clarity of MS^2^ spectra by allowing fragmentation of only the ions confining within a quadrupole transmission profile. However, it loses a portion of the beam not confining to a quadrupole transmission window and hence, results in decreased overall sensitivity. Ion mobility separation based DIA, on the other hand, operates under a principle of preion mobility separation ion accumulation and subsequent release and hence, does not suffer from the ion loss of the quadrupole-based methods. As fragments can only exist when a precursor is present and fragments are inheriting the same drift time as the precursor due to the fact that mobility separation takes place before the fragmentation, it has been reported that ion mobility separation achieves a similar type of MS^2^ clarity using the alignment of drift times and chromatographic profile of a precursor and fragments (HDMS^E®^/PASEF^®^) [26,27]. Implementation of enhanced DIA methods would allow for even faster gradients and is worth further investigation.

A cut-off filter (3 kDa) was selected for sample preparation due to the unclear interaction of shorter cheese peptides with reversed-phase solid-phase extraction. In our work, we observed bias towards shorter peptides compared to Taivosalo et al. [14]. This bias could have been caused by either a natural bias of the given system towards shorter peptides, a decreased loss of shorter peptides due to not using of reverse-phase solid-phase extraction, or an increased loss of longer peptides due to the use of 3 kDa cut-off filter. As the overall number of peptides identified was lower than anticipated the use of 3 kDa cut-off filter should be further reviewed for its performance against conventional reversed-phase solid-phase extraction methods.

## 5. Conclusions

A rapid method was developed and successfully applied to the cheese peptidomics studies. The study allowed to indicate differences in cheese ripening caused due to the use of different starter cultures. Further methodology development is possible via the deployment of enhanced DIA approaches. Enhanced transmission of shorter peptides presents additional interest for future studies due to recorded bioactivity and sensory effects.

## Supplementary Materials

The following are available online at https://www.mdpi.com/2304-8158/9/8/979/s1, Figure S1: Summed peptide intensities of Cheese 1, 2 and 3 at the day 90, Figure S2: Peptide length distribution across 3 cheeses at the day 90, Figure S3: Overlay of Base Peak Intensity and Extracted Ion Chromatogram for narrowest peak corresponding to an identified peptide.
