# Supplementary material for: Application of the UHPLC-DIA-HRMS Method for Determination of Cheese Peptides
Source: Foods. 2020 Jul 23;9(8):979. doi: 10.3390/foods9080979 (PMC7466222; doi:10.3390/foods9080979)
Supplement: Supplementary file 1 [file foods-09-00979-s001.pdf]

supplementary：

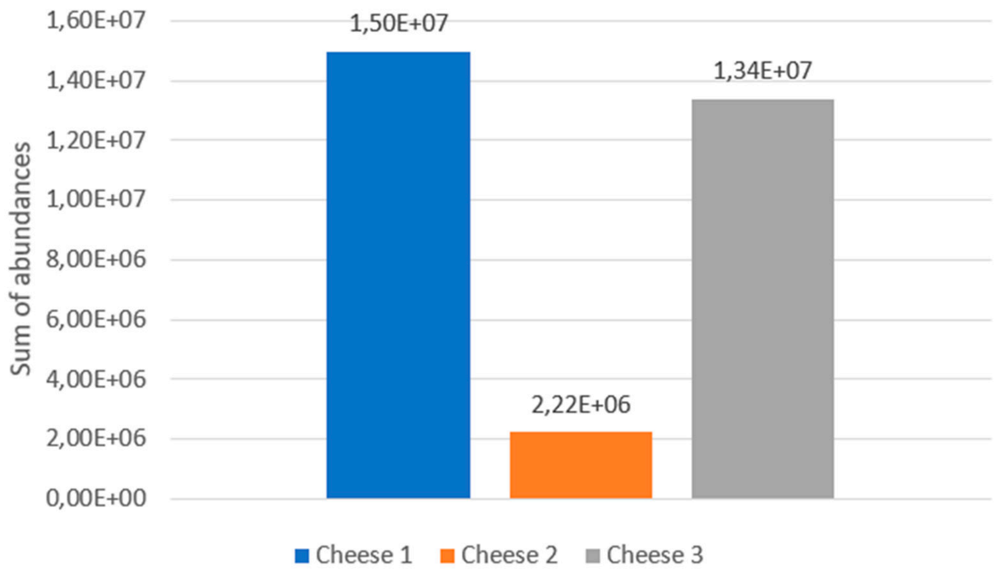

Figure 1. Summed peptide intensities of Cheese 1, 2 and 3 at the day 90.

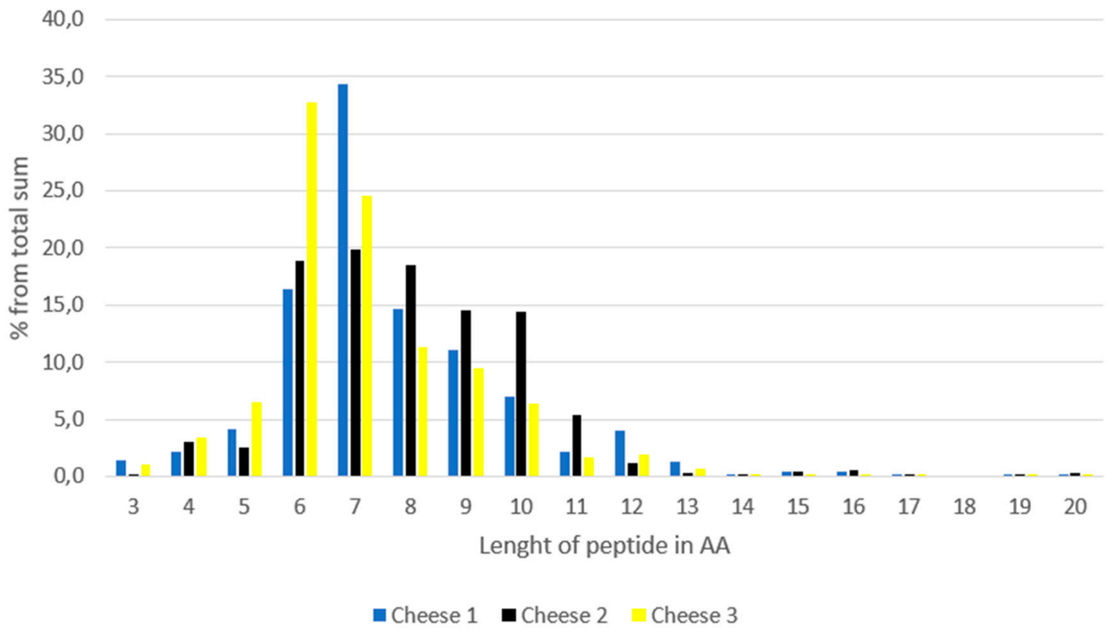

Figure 2. Peptide length distribution across 3 cheeses at the day 90.

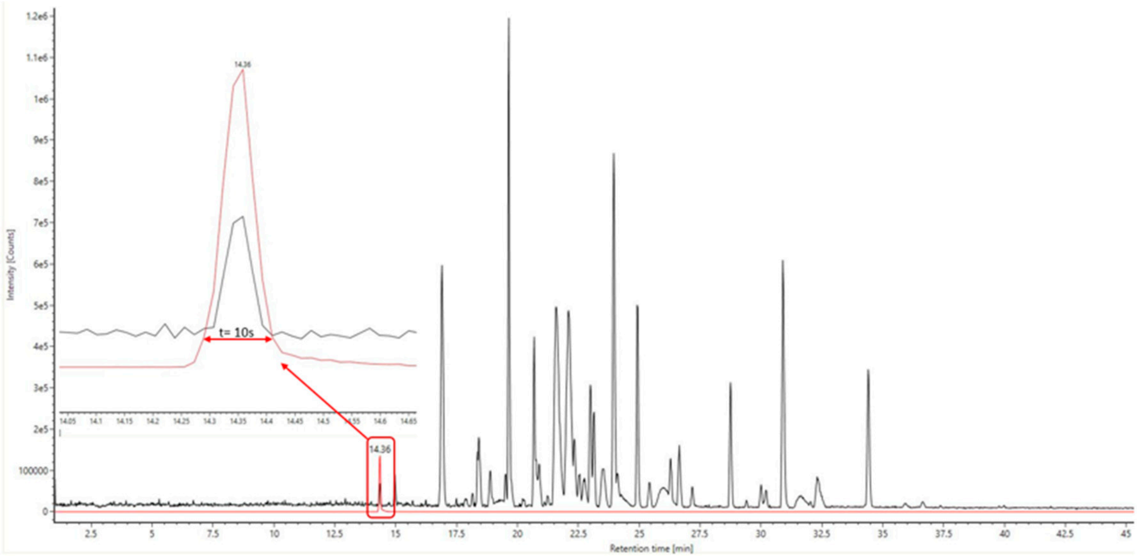

Figure 3. Overlay of Base Peak Intensity and Extracted Ion Chromatogram for narrowest peak corresponding to an identified peptide.
